# Hypoxic breathing produces more intense hypoxemia in elderly women than in elderly men

**DOI:** 10.3389/fphys.2022.989635

**Published:** 2022-10-26

**Authors:** Jinfeng Zhao, Yanfeng Ding, Geoffrey P. Kline, Zhengyang Zhou, Robert T. Mallet, Xiangrong Shi

**Affiliations:** ^1^ Departments of Pharmacology and Neuroscience, School of Biomedical Sciences, University of North Texas Health Science Center, Fort Worth, TX, United States; ^2^ School of Physical Education, Shanxi University, Taiyuan, China; ^3^ Internal Medicine, University of North Texas Health Science Center, Fort Worth, TX, United States; ^4^ Biostatistics and Epidemiology, School of Public Health, University of North Texas Health Science Center, Fort Worth, TX, United States; ^5^ Physiology and Anatomy, School of Biomedical Sciences, University of North Texas Health Science Center, Fort Worth, TX, United States

**Keywords:** aging, chemoreflex, heart rate (HR), oxygen dissociation, oxygen extraction fraction (OEF), ventilation

## Abstract

**Background:** Brief hypoxic exposures are increasingly applied as interventions for aging-related conditions. To optimize the therapeutic impact of hypoxia, knowledge of the sex-related differences in physiological responses to hypoxia is essential. This study compared hypoxia-induced hypoxemic responses in elderly men and women.

**Methods**: Seven elderly men (70.3 ± 6.0 years old) and nine women (69.4 ± 5.5 years old) breathed 10% O_2_ for 5 min while arterial (SaO_2_; transcutaneous photoplethysmography) and cerebral tissue O_2_ saturation (ScO_2_; near-infrared spectroscopy), ventilatory frequency, tidal volume, minute-ventilation, and partial pressures of end-tidal O_2_ (P_ET_O_2_) and CO_2_ (mass spectrometry) were continuously monitored. Cerebral tissue oxygen extraction fraction (OEF) equaled (SaO_2_–ScO_2_)/SaO_2_.

**Results:** During 5 min hypoxia SaO_2_ fell from 97.0 ± 0.8% to 80.6 ± 4.6% in the men and from 96.3 ± 1.4% to 72.6 ± 4.0% in the women. The slope ΔSaO_2_/min was steeper in the women than the men (−4.71 ± 0.96 vs. −3.24 ± 0.76%/min; *p* = 0.005). Although SaO_2_ fell twice as sharply per unit decrease in P_ET_O_2_ in the women than the men (−1.13 ± 0.11 vs. −0.54 ± 0.06%/mmHg; *p* = 0.003), minute-ventilation per unit hypoxemia increased less appreciably in the women (−0.092 ± 0.014 vs. −0.160 ± 0.021 L/min/%; *p* = 0.023). OEF fell with hypoxia duration in the women, but remained stable in the men.

**Conclusion:** During 5 min hypoxic breathing, elderly women experience more intense hypoxemia and reduced chemoreflex sensitivity vs. their male counterparts, which may lower OEF stability in women despite augmented O_2_ dissociation from hemoglobin during hypoxia. These sex-related differences merit attention when implementing brief hypoxic exposures for therapeutic purposes.

## Introduction

The differences in oxygen carrying capacity in men vs. women are well recognized. Women have smaller lung volumes (Mead 1980; Sheel, et al., 2004), lower hematocrits and blood hemoglobin (Hb) contents (Tilling, et al., 2013; Tzounakas, et al., 2021), and smaller plasma and blood volumes (Diaz-Canestro, et al., 2022a; Diaz-Canestro, et al., 2022b). Collectively, these factors predict lower cardiorespiratory function and aerobic capacity in women vs. men (Harms 2006; Diaz-Canestro, et al., 2022a). Moreover, exercise induced arterial hypoxemia is more prevalent in women than men (Harms, et al., 1998; Richards, et al., 2004), which may be ascribed to less robust acute ventilatory responses to hypoxia in women (Guenette, et al., 2004). Cardiac baroreflex responses were attenuated in middle-aged women vs. age-matched men, while estrogen replacement in women abolished the sex difference in these responses (Huikuri, et al., 1996). Female sex hormones also dampen apnea-induced neurogenic vasoconstriction (Patel, et al., 2014). However, scant data are available regarding the sex-related differences in chemoreflex-mediated cardiac or ventilatory responses to hypoxemia, which could persist even after sex hormones have subsided in older women.

Intermittent hypoxic (IH) training involving cyclic ventilation with moderately hypoxic gas alternated with room air breathing, has been increasingly adopted to treat various age-related pathological conditions. IH training proved beneficial in patients with coronary artery disease (Burtscher, et al., 2004), chronic obstructive pulmonary disease (Burtscher, et al., 2009; Haider, et al., 2009), hypertension (Lyamina, et al., 2011), and mild cognitive impairment (Bayer, et al., 2017b; Serebrovska, et al., 2019; Wang, et al., 2020). Moreover, IH programs alternating moderately hypoxic and hyperoxic ventilation combined with physiotherapy or physical activity have found to improve cognitive performance and physical function and health in elderly adults (Bayer, et al., 2017a; Bayer, et al., 2017b; Bayer, et al., 2019; Serebrovska, et al., 2019; Behrendt, et al., 2022). In contrast, intense IH imposed by obstructive sleep apnea (hypoxia combined with hypercapnia) is an established risk factor for heart disease, stroke and cognitive impairment. Because the intensities of hypoxia-induced hypoxemia, tissue hypoxia and its related physiological responses are pivotal determinants of the benefits vs. detriments of hypoxic interventions (Navarrete-Opazo and Mitchell 2014), knowledge of sex differences in cardio-respiratory responses to hypoxia are indispensable to optimize the therapeutic efficacy of IH training.

This study compared the changes in arterial O_2_ saturation (SaO_2_), cerebral tissue oxygenation (ScO_2_) and cardiac and ventilatory responses during moderate hypoxic exposure in elderly men vs. women. The subjects breathed 10% O_2_ for 5 min, resulting in a mild to moderate hypoxemia which is well-tolerated (Zhang, et al., 2010; Liu, et al., 2017; Liu, et al., 2020) and improves cerebrovascular and cardioventilatory function in healthy adults (Zhang, et al., 2014; Zhang, et al., 2015; Wang, et al., 2020). Because women have lower aerobic capacity, cardiorespiratory function, acute ventilatory responses to hypoxia, and cardiac baroreflex responses than their male counterparts, this study tested the hypothesis that elderly women will experience more intense hypoxemia than elderly men when breathing moderately hypoxic air.

## Materials and methods

### Participants

Sixteen elderly men (n = 7) and women (n = 9) free of cardiorespiratory, metabolic and renal disease voluntarily participated in the study after providing written informed consent and passing a physical screening with arterial blood pressure below 140/90 mmHg. The men and women had similar body mass indices, although height and body surface area estimated from the DuBois formula (DuBois and DuBois, 1916) were significantly smaller in the women than men (Table 1). The study protocols were reviewed and approved by the Institutional Review Board for the Protection of Human Subjects at the University of North Texas Health Science Center (IRB Project #2016-070).

**TABLE 1:** Physical characteristics and baseline variables of the subjects.

	Men (n = 7)	Women (n = 9)	*p* Value
Physical characteristics			
Age (year)	70.3 ± 6.0	69.4 ± 5.5	0.777
Weight (kg)	81.4 ± 14.0	74.4 ± 6.6	0.276
Height (m)	1.75 ± 0.08	1.62 ± 0.05	0.002
BMI (kg/m^2^)	26.5 ± 3.7	28.5 ± 2.3	0.206
BSA (m^2^)	1.96 ± 0.23	1.75 ± 0.10	0.038
Baseline variables			
SaO_2_ (%)	97.0 ± 0.8	96.3 ± 1.4	0.253
ScO_2_ (%)	70.1 ± 2.7	67.9 ± 5.2	0.339
*f*_Br_ (cycle/min)	12.7 ± 3.3	14.2 ± 3.2	0.379
Tidal volume (L)	0.62 ± 0.24	0.45 ± 0.09	0.118
Ventilation (L/min)	7.44 ± 1.71	6.43 ± 2.01	0.307
P_ET_O_2_ (mmHg)	107 ± 4	104 ± 4	0.131
P_ET_CO_2_ (mmHg)	40.9 ± 2.5	42.9 ± 4.4	0.308
Heart rate (min^−1^)	63 ± 11	74 ± 12	0.069
SBP (mmHg)	124 ± 8	134 ± 5	0.013
DBP (mmHg)	69 ± 8	79 ± 9	0.043
MAP (mmHg)	87 ± 7	97 ± 7	0.016
OEF	0.277 ± 0.033	0.295 ± 0.055	0.470

Mean values ±SD. BMI: body mass index; BSA: body surface area, estimated according to DuBois formula. SaO_2_: arterial oxygen saturation; ScO_2_: cerebral tissue oxygenation; *f*_Br_: breathing rate; P_ET_O_2_: partial pressure of end tidal O_2_; P_ET_CO_2_: partial pressure of end tidal CO_2_; SBP: systolic blood pressure; DBP: diastolic blood pressure; MAP: mean arterial pressure; OEF: oxygen extraction fraction = (SaO_2_—ScO_2_)/SaO_2_.

### Hypoxia protocol

All subjects breathed poikilocapnic hypoxic air containing 10% O_2_. Before each experiment, a mass spectrometer gas analyzer (Perkin-Elmer, 1100 Medical Gas Analyzer, St Louis, Missouri) was calibrated with room air and medical gas (Instrumentational Laboratory, Lexington, MA) containing 10% O_2_ and 5% CO_2_ (balance N_2_). After calibration, the gas analyzer confirmed the hypoxic gas contained 10 ± 0.2% O_2_ (balance N_2_). All measurements were made with the subject wearing a disposable air-cushioned facemask (VacuMed, Ventura, CA) while resting in the supine position. After 10 min equilibration, baseline (pre-hypoxic) measurements were collected for approximately 3 min, and then normobaric, poikilocapnic hypoxia was applied for 5 min. No discomfort, distress, or dizziness was reported by any subjects or observed by the investigators, either before, during or after hypoxia. Ambient conditions were maintained throughout testing with barometric pressure 735–745 mmHg, relative humidity 50–58% and room temperature 24 ± 1°C.

### Measurements

Measurements of O_2_, CO_2_ and ventilatory and cardiovascular function were described previously (Zhang, et al., 2010; Zhang, et al., 2014; Liu, et al., 2020) and are summarized here. Systemic arterial O_2_ saturation (SaO_2_) was measured by a transcutaneous sensor (TOSCA 500, Radiometer America Inc., Westlake, OH, United States) applied to the right earlobe. This measurement yielded SaO_2_ values essentially identical to values obtained from the finger using BIOPAC OXY100C (lab observation). Cerebral tissue O_2_ saturation (ScO_2_) of the prefrontal cortex was monitored by near-infrared spectroscopy with a sensor (Somanetics, 5100 INVOS Cerebral Oximeter, Troy, MI) placed on the right forehead. Cerebral tissue oxygen extraction fraction (OEF) was taken as (SaO_2_—ScO_2_)/SaO_2_.

Breath-by-breath inspired and expired fractions of O_2_ and CO_2_ were continuously monitored by mass spectrometry. Gas was sampled *via* a tubing embedded in the inlet of a Universal Ventilation Meter (UMV VacuMed, Ventura, CA), which recorded breath-by-breath ventilatory frequency (*f*_Br_) and tidal volume (V_T_). Minute ventilation equaled V_T_ • *f*_Br_. Partial pressures of O_2_ and CO_2_ in end-tidal gas (P_ET_O_2_ and P_ET_CO_2_) were calculated by multiplying ambient barometric pressure by the expired fractions of O_2_ and CO_2_, respectively. Heart rate (HR) was monitored by standard limb lead II electrocardiography. Systolic and diastolic blood pressures (SBP and DBP) were measured by double finger cuffs placed on the proximal phalanges of the left index and middle fingers (CNAP 500, Graz, Austria). Mean arterial pressure (MAP) was calculated as 1/3 of SBP plus 2/3 of DBP. Analog data were continuously digitized at 250 Hz by a computer interfaced with a data acquisition system (MP150 BIOPAC, Santa Barbara, CA).

### Data analyses

Baseline (i.e., 0 min hypoxia) variables were averaged from 60 s of continuous data collected immediately before initiating hypoxic breathing. During 5 min hypoxia, data from the last 30s of each minute were averaged to represent the minute-by-minute data (Zhang, et al., 2010; Liu, et al., 2017; Liu, et al., 2020). All ventilatory, cardiovascular, SaO_2_ and ScO_2_ data during hypoxic exposure passed the Shapiro-Wilk normality test, except breathing frequency in the female group. Demographic and baseline variables in male vs. female subjects were compared using two-sample *t*-test with Statistical Analysis System (SAS) software (Version 9.4, Cary, NC). Changes in SaO_2_ and ScO_2_ vs. hypoxia duration, i.e. slope, were determined using linear regression models in SAS. The effect-size index of both SaO_2_ and ScO_2_ slopes was estimated through the partial omega squared ( $\omega^{2}$ ) statistic (Olejnik and Algina 2003). Two-factor ANOVA (PROC ANOVA) was applied to assess the impact of sex (group factor) and hypoxic duration (hypoxic factor) on SaO_2_, ScO_2,_ ventilatory and cardiovascular variables. *Post-hoc* analysis of the group difference was performed if ANOVA detected statistical significance. The association of SaO_2_ with P_ET_O_2_ was examined as an index of O_2_-hemoglobin dissociation. Although the relationship between SaO_2_ and P_ET_O_2_ was not simply linear or sigmoidal (Zhang, et al., 2014), the approximately linear portion of SaO_2_ vs. P_ET_O_2_, i.e., the peak SaO_2_/P_ET_O_2_ slope, was evaluated to provide an index of O_2_ unloading from hemoglobin during hypoxic exposure (Zhang, et al., 2010; Zhang, et al., 2014). The slopes of minute ventilation and HR vs. SaO_2_ and P_ET_O_2_ during hypoxia were plotted as measures of chemoreflex sensitivity to hypoxemia. Covariate analysis (ANCOVA) was conducted to test the differences in the slopes between men and women. Result values are reported as group mean ± standard deviation (SD). *p* values ≤0.05 were taken statistical significance.

## Results

### Baseline variables

Baseline SaO_2_, ScO_2_, P_ET_O_2_, P_ET_CO_2_ and ventilatory function variables did not differ significantly between the elderly male and female subjects (Table 1). However, systolic, diastolic and mean arterial pressures were higher in the women than the men, and HR showed a strong trend toward higher values in the women.

### SaO_2_ and ScO_2_ during hypoxic exposure

**FIGURE 1:**
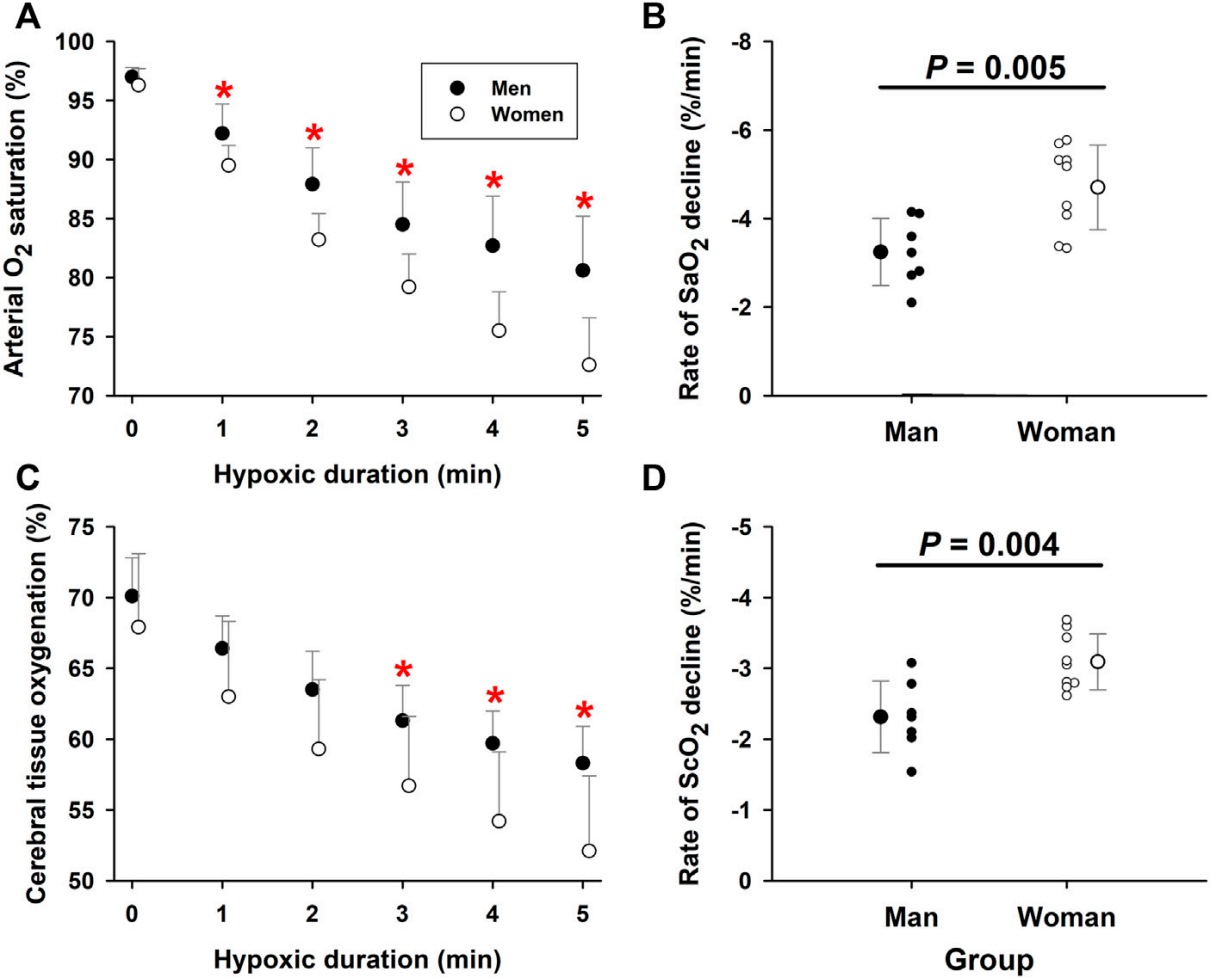
*Arterial O*_*2*_ *saturation and cerebral tissue oxygenation during moderate hypoxia in elderly men* vs*. women*. Panel **(A)**: Arterial O_2_ saturation (SaO_2_) fell more rapidly in elderly women than men during 5-min moderate hypoxia. Panel **(B)**: The individual and group rates of SaO_2_ decline during 5-min hypoxia were greater in the elderly women than men (unpaired *t*-test). Panel **(C)**: ScO_2_ fell with hypoxia duration to a greater extent in elderly women than men. Panel **(D)**: ScO_2_ fell more rapidly in elderly women than elderly men during 5-min hypoxia (unpaired *t*-test). *Post-hoc analysis: *p* < 0.05 vs. values in women. Mean values ±SD. Panels **(B)** and **(D)** also show values in individual subjects.

Arterial O_2_ saturation fell progressively with the duration of hypoxia exposure in both elderly men (−3.25 ± 0.31%/min, R^2^ = 0.96, *p* = 0.001) and women (−4.70 ± 0.40%/min, R^2^ = 0.97, *p* = 0.001). During the fifth minute of hypoxia, SaO_2_ was 80.6 ± 4.6% in the men and 72.6 ± 4.0% in the women, respectively. ANCOVA revealed a significant interaction of hypoxic duration x group factor (*p* = 0.021), indicating a significant difference in the slopes of the two groups (Figure 1A). The rate of SaO_2_ decline, was significantly greater (*p* = 0.005) in the elderly women (−4.71 ± 0.96%/min, n = 9) than men (−3.24 ± 0.76%/min, n = 7) (Figure 1B), indicating 5 min exposure to 10% O_2_ produced more rapid intensification of arterial hypoxemia in the elderly women. ScO_2_ paralleled the hypoxemia. During the 5-min hypoxia, ScO_2_ values fell from 67.9 ± 5.2% to 52.1 ± 5.3% in the women and from 70.1 ± 2.7% to 58.3 ± 2.6% in the men (Figure 1C). ANCOVA revealed a more rapid ScO_2_ decline (*p* = 0.052) in the women (−3.09 ± 0.25%/min, R^2^ = 0.97, *p* = 0.001) than men (−2.32 ± 0.22%/min, R^2^ = 0.96, *p* = 0.001). The slope ΔScO_2_/min was steeper (*p* = 0.004) in the elderly women (−3.09 ± 0.39%/min, n = 9) than men (−2.31 ± 0.50%/min, n = 7); thus, the elderly women experienced more intense cerebral tissue hypoxia during hypoxic breathing (Figure 1D). The partial Omega squared ( $\omega^{2}$ ) statistic equaled 0.383 for SaO_2_ slope (F_1,14_ = 10.92; *p* = 0.005; adjusted R^2^ = 0.398) and 0.407 for ScO_2_ slope (F_1,14_ = 11.98; *p* = 0.004; adjusted R^2^ = 0.423). These effect sizes of SaO_2_ and ScO_2_ data indicated that sex related differences accounted for about 40% of the variance of the outcomes.

### Respiratory and cardiovascular responses during hypoxic exposure

**FIGURE 2:**
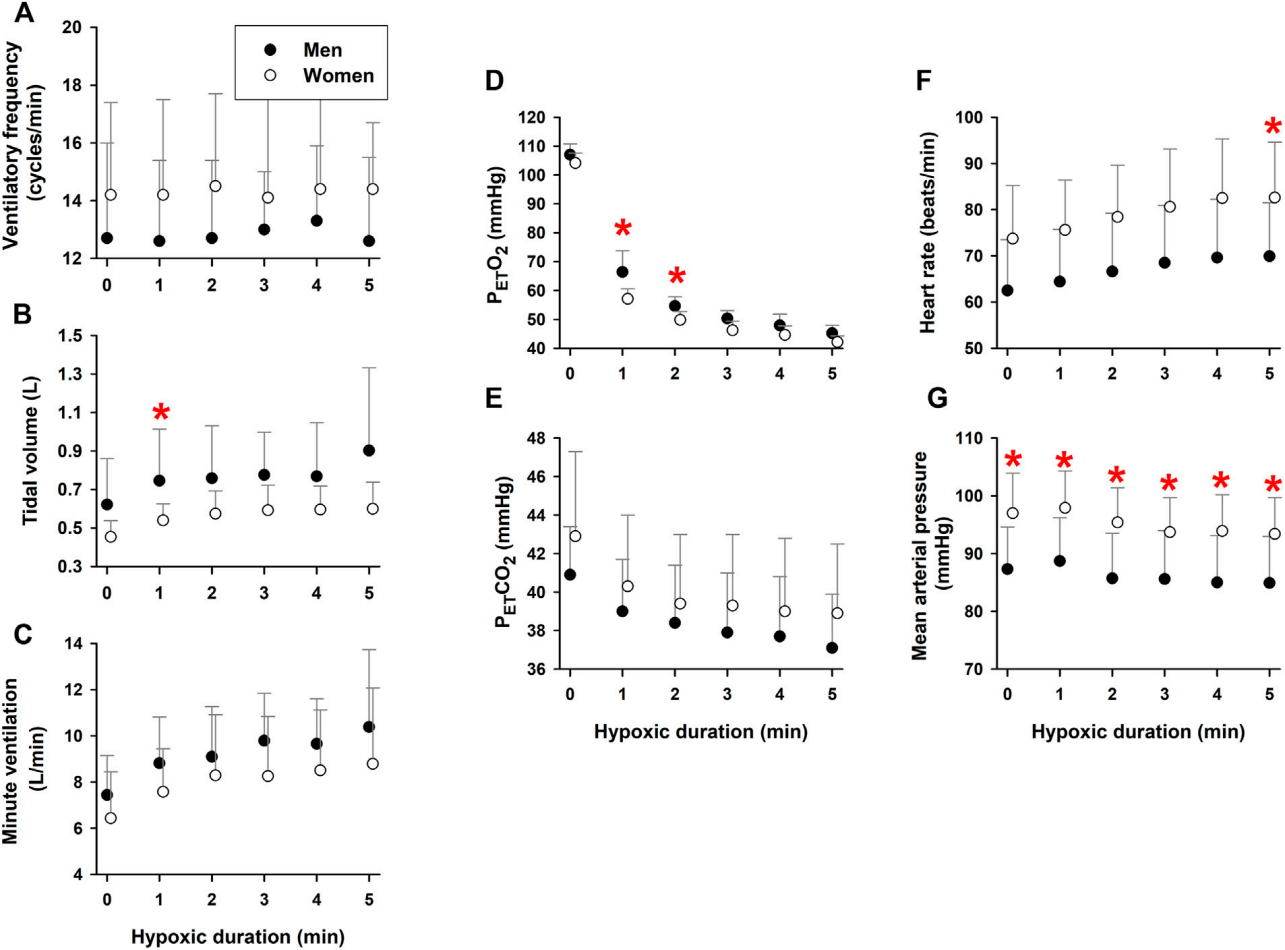
Respiratory and cardiovascular responses during 5-min hypoxia. Ventilatory frequency did not change [Panel **(A)**], while tidal volume [Panel **(B)**] and, thus, minute-ventilation [Panel **(C)**] increased during hypoxia in elderly men and women. The women had higher ventilatory frequencies and lower tidal volumes and minute ventilation than the men. P_ET_O_2_ [Panel **(D)**] and P_ET_CO_2_ [Panel **(E)**] fell during hypoxia. The women [had lower P_ET_O_2_ and higher P_ET_CO_2_ values than the men. Hypoxic exposure increased heart rate [Panel **(F)**], without affecting mean arterial pressure [Panel **(G)**]. Heart rate and mean arterial pressure were higher in the elderly women than men. *Post-hoc analysis: *p* < 0.05 vs. values in women. Mean values ±SD.

Both V_T_ (time factor *p* = 0.003) and minute ventilation (time factor *p* = 0.001) increased during hypoxia, as expected. Ventilatory frequency (*f*_Br_) did not change during 5-min hypoxia (time factor *p* = 0.486) (Figure 2A), but remained higher in the women than men (group factor *p* = 0.001). Since *f*_Br_ was unchanged, the increased minute ventilation could be ascribed to the hypoxemia-elicited increase in V_T_ (Figures 2B,C). Both V_T_ and minute ventilation were lower in the women than the men (group factor *p* = 0.001 for V_T_ and *p* = 0.003 for minute ventilation).

P_ET_O_2_ fell as systemic hypoxemia intensified (time factor *p* = 0.003) during 5-min hypoxia (Figure 2D). P_ET_CO_2_ also fell during hypoxia (time factor *p* = 0.008), due to increased minute ventilation (Figure 2E). P_ET_O_2_ was lower (group factor *p* = 0.012) and P_ET_CO_2_ was greater (group factor *p* = 0.001) in the women vs. the men. Heart rate increased during hypoxia (time factor *p* = 0.015) (Figure 2F), but mean arterial pressure did not (time factor *p* = 0.205), indicating that the hypoxemia elicited tachycardiac but not hypertensive responses (Figure 2G). Both HR and MAP were greater (group factor *p* = 0.001) in the women than men throughout hypoxia.

### Sex-related differences in cardioventilatory responses to hypoxemia

**FIGURE 3:**
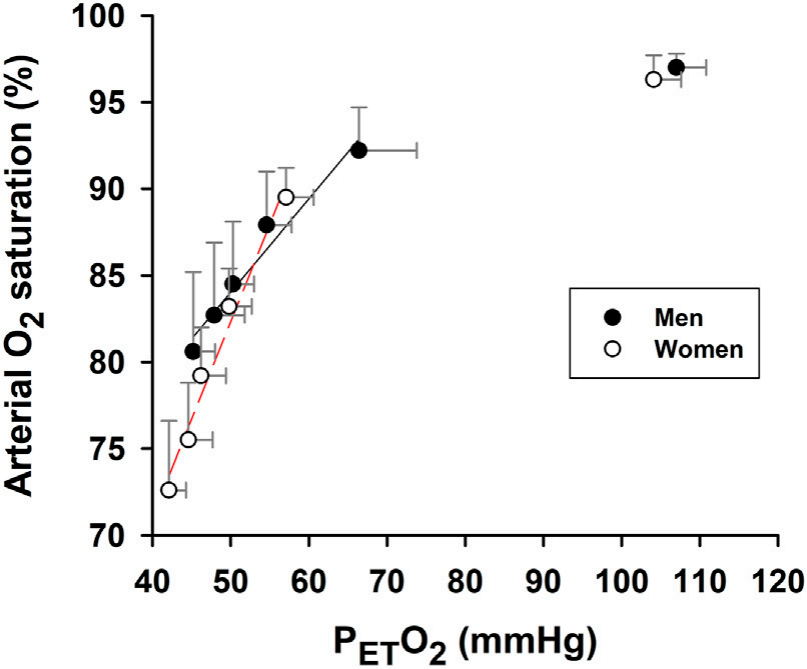
Association of arterial O_2_ saturation and partial pressure of end-tidal O_2_. Slope of SaO_2_ vs. end-tidal partial pressure O_2_ (P_ET_O_2_) during 5-min hypoxia was significantly steeper in women than men. Baseline (pre-hypoxia) data (upper right) are not included in the linear regressions (solid and broken lines) or slope comparison. Symbols represent mean values ±SD at each min of the 5-min hypoxia exposure.

**FIGURE 4:**
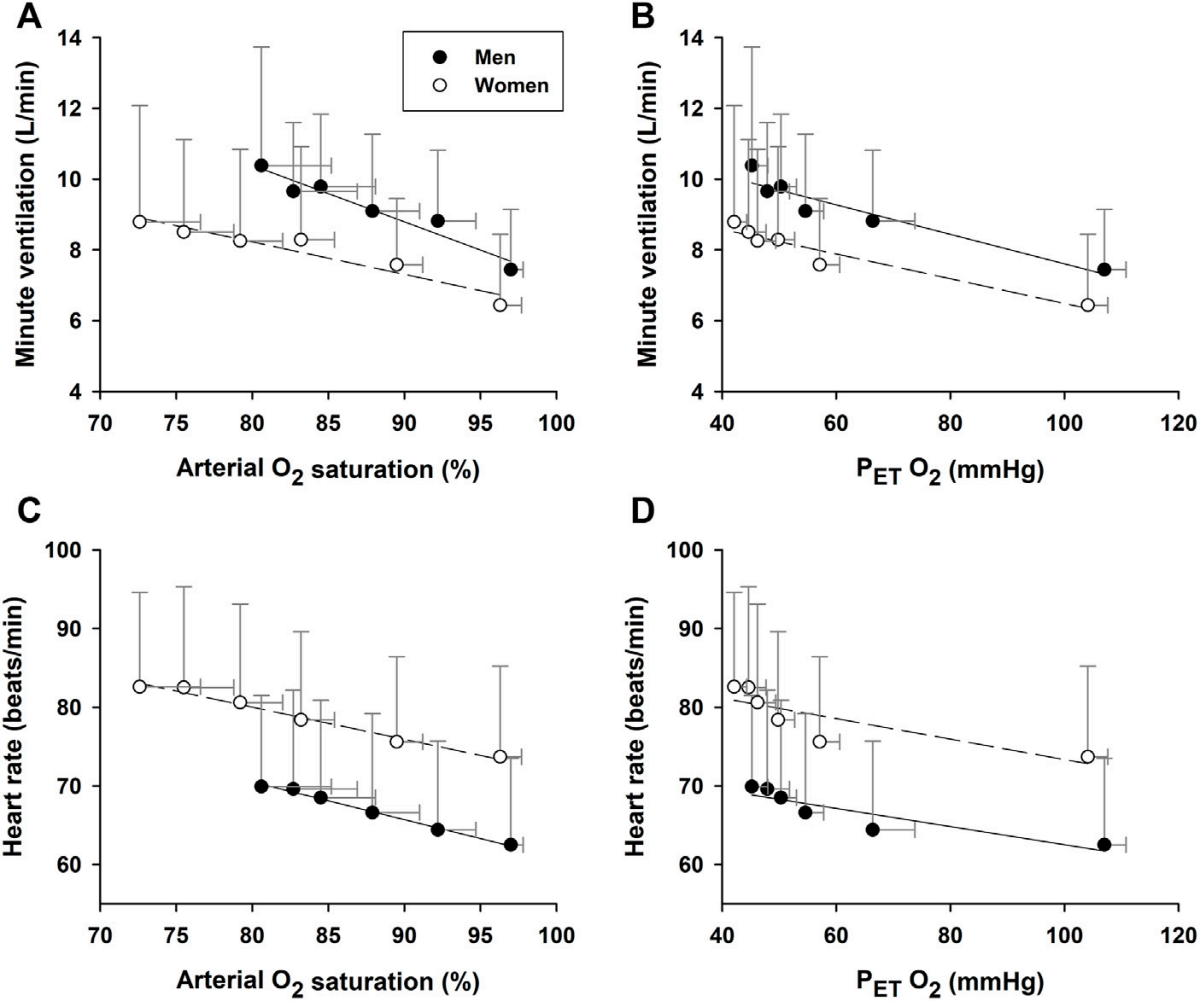
Associations of minute ventilation and heart rate vs. arterial O_2_ saturation. The increase of minute ventilation per unit decrease in arterial O_2_ saturation during hypoxic exposure [Panel **(A)**] was attenuated in the elderly women vs. men. However, the slopes of minute ventilation per unit decrease in P_ET_O_2_ [Panel **(B)**] were not significantly different between the two groups. Neither the increase in heart rate per unit SaO_2_ decrease [Panel **(C)**] nor the heart rate decrease per unit decrease in P_ET_O_2_ [Panel **(D)**] differed between the groups.

**FIGURE 5:**
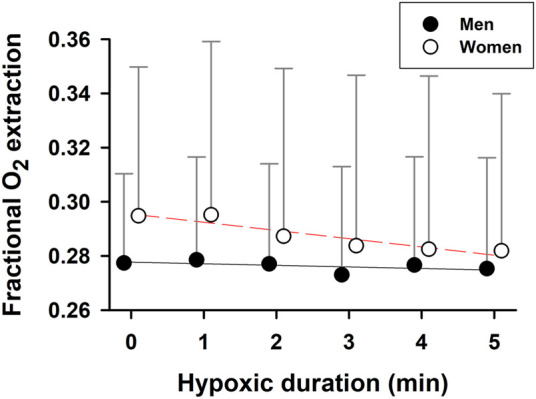
Cerebral tissue fractional oxygen extraction during hypoxia. Cerebral tissue fractional O_2_ extraction declined with hypoxic time in the elderly women, but was stable over 5-min hypoxia in the elderly men. Mean values ±SD.

During hypoxic exposure, the slope of SaO_2_ vs. P_ET_O_2_ was doubled (*p* = 0.003) in the women (1.125 ± 0.106%/mmHg, R^2^ = 0.97, *p* = 0.002) vs. men (0.539 ± 0.062%/mmHg, R^2^ = 0.95, *p* = 0.003) (Figure 3). This apparent enhancement of O_2_-hemoglobin dissociation indicated that the elderly women reached the steeper portion of the O_2_-hemoglobin dissociation curve earlier in hypoxia than the men. Increases in minute ventilation could be ascribed to decreases in SaO_2_ in both the women (−0.092 ± 0.014 L/min/%; R^2^ = 0.90, *p* = 0.003) and men (−0.160 ± 0.020 L/min/%; R^2^ = 0.95, *p* = 0.002) (Figure 4A). However, the Δminute ventilation/ΔSaO_2_ slope was shallower (*p* = 0.023) in the women than men during hypoxic exposure, suggesting lower sensitivity of the chemoreflex-mediated ventilatory compensation in elderly women. On the other hand, the slope of minute ventilation vs. P_ET_O_2_ (Figure 4B) was not different (*p* = 0.441) in the women (−0.035 ± 0.005 L/min/mmHg, R^2^ = 0.91, *p* = 0.002) vs. men (−0.042 ± 0.007 L/min/mmHg, R^2^ = 0.88, *p* = 0.003). Although the ΔHR/ΔSaO_2_ slope tended to be shallower in the women (-0.409 ± 0.027 bpm/%; R^2^ = 0.98, *p* < 0.001) vs. men (−0.480 ± 0.022 bpm/%; R^2^ = 0.99, *p* < 0.001) (Figure 4C), the difference was not statistically significant (*p* = 0.107). The ΔHR/ΔP_ET_O_2_ slope was not different (*p* = 0.689) in the women (−0.131 ± 0.043 bpm/mmHg, R^2^ = 0.62, *p* = 0.040) vs. the men (−0.109 ± 0.025 bpm/mmHg, R^2^ = 0.78, *p* = 0.012) (Figure 4D). Cerebral tissue OEF in the women fell with increasing hypoxia duration (slope −0.0030 ± 0.0006 fraction/min; R^2^ = 0.88, *p* = 0.006), while in the men, OEF did not change during hypoxia (*p* = 0.248) (Figure 5).

## Discussion

This study is the first to demonstrate sex differences in hypoxia-induced arterial hypoxemia and cerebral tissue hypoxia in elderly adults, where 5-min exposure to 10% O_2_ produced more precipitous declines in arterial O_2_ saturation and cerebral tissue oxygenation in women vs. men. In general, women have smaller physiques and, thus, lower blood volume and hemoglobin mass than their male counterparts (Falz et al., 2019; Diaz-Canestro et al., 2022a; Diaz-Canestro et al., 2022b); consequently, vascular PaO_2_ and O_2_ content are likely to fall more rapidly in women than men during hypoxia. Indeed, in this study O_2_-hemoglobin dissociation at a given decrease in P_ET_O_2_ was greater in women than men exposed to the same intensity (10% O_2_) and duration (5 min) of hypoxic breathing. Furthermore, the increase in minute ventilation per unit decrease in SaO_2_ was smaller in the women, indicating less robust chemoreflex-mediated ventilatory compensation than in men. Consequently, cerebral tissue OEF progressively fell with hypoxic duration in the elderly women, but remained constant during hypoxia in the elderly men.

The mechanisms producing the more severe hypoxia-induced arterial hypoxemia in elderly women are not fully understood. We postulated that the lower oxygen carrying capacity and cardiorespiratory function in women could contribute to more intense hypoxemia during hypoxic exposure. Underlying factors could include lower blood hemoglobin content (Tilling, et al., 2013; Tzounakas, et al., 2021), lower circulating capacity, i.e., total blood volume (Diaz-Canestro, et al., 2022a; Diaz-Canestro, et al., 2022b), and smaller reservoir, i.e., body physique or surface area (see Table 1) in women. Collectively, these factors would cause blood O_2_ content to fall more rapidly in women than men at a given hypoxia intensity, producing a more severe arterial hypoxemia at the same hypoxic intensity and duration. A diminished chemoreflex-mediated ventilatory response in the elderly women vs. their age-matched male counterparts also may have contributed to the more intense hypoxemia during 10% O_2_ breathing in women vs. men.

We previously reported a shift toward decreased hemoglobin O_2_-binding affinity during hypoxia, a phenomenon which intensified with repeated hypoxic exposures (Zhang, et al., 2010; Zhang, et al., 2014). Enhanced O_2_-hemoglobin dissociation augments O_2_ release to O_2_-consuming tissues in proportion to the duration and number of hypoxic exposures, potentially offsetting the decreased arterial O_2_ content. However, increased O_2_ unloading also exacerbates arterial hypoxemia by depleting oxygenated hemoglobin. This mechanism may underlie the greater exercise-induced arterial hypoxemia in exercise trained young adults vs. age-matched, sedentary men and women (Guenette, et al., 2004). Our data indicate that the rate of SaO_2_ decrease per unit decrease in P_ET_O_2_ was appreciably greater in elderly women vs. men (Figure 3). This phenomenon was likely a pivotal contributor to the greater hypoxia-induced hypoxemia in the elderly women in this study.

Evidence for sex-related differences in ventilatory responses to hypoxia remains inconclusive (Guenette, et al., 2004). Some studies reported no sex difference in ventilatory response to acute normobaric poikilocapnic or isocapnic hypoxia (10% O_2_) in young adults (Rispen, et al., 2017), yet others have shown less robust ventilatory responses to mild normobaric hypoxia (15% O_2_) associated with more intense hypoxemia in women (Camacho-Cardenosa, et al., 2022). In the present study, the ventilation increase per unit hypoxemia (i.e., decrease in SaO_2_) was significantly lower in the elderly women than men (Figure 4A: −0.10 vs. −0.16 L/min/%, *p* = 0.023). This attenuated chemoreflex-mediated ventilatory response could not be ascribed to counteraction by hyperventilation-induced hypocapnia, because P_ET_CO_2_ was persistently higher in the women than men (Figure 2E). The tachycardic response per unit hypoxemia was not significant between the two groups (Figure 4B: −0.41 bpm/% in women vs. −0.48 bpm/% in men; *p* = 0.107).

During hypoxia, cerebral tissue fractional O_2_ extraction is increased or maintained in young adults (Liu, et al., 2017), whereas skeletal muscle OEF declines with exposure time (Zhang, et al., 2010). These tissue-specific differences in OEF, which may favor greater O_2_ release from hemoglobin in the brain vs. muscles, should serve to protect the brain, which is utterly dependent on O_2_ for ATP production. In this study, cerebral tissue OEF remained constant in elderly men, but fell in elderly women during hypoxia (Figure 5). The decreased OEF in the women was associated with more intense arterial hypoxemia combined with a diminished ventilatory response during hypoxia, could destabilize O_2_ delivery to the brain in elderly women vs. men.

This study confirmed that increases in minute ventilation in both elderly men and women during hypoxic breathing were predominantly driven by increases in tidal volume. Deeper breathing during hypoxia may lower thoracic and jugular venous pressures, thereby facilitating cerebral venous *outflow* to accommodate the increased cerebral arterial *inflow* in response to hypoxia-induced cerebral vasodilation (Liu, et al., 2017). This study further confirmed that moderate hypoxia-induced hypoxemia caused no hypertensive responses in elderly men and women. Although hypoxic exposure increased HR in both sexes, there was no increase in MAP associated with the tachycardic response. Since cardiac output could increase with the increased heart rate, MAP stability during hypoxia suggests that active vasodilation may have counteracted hypoxia-induced sympathoexcitation or increased heart rate.

### Study limitations and perspectives

Study limitations include using P_ET_O_2_ instead of partial pressure of dissolved O_2_ in arterial blood (PaO_2_) to evaluate O_2_-hemoglobin dissociation during hypoxic exposure. Nonetheless, breath-by-breath continuously measured P_ET_O_2_ should change in lockstep with alveolar PO_2_ (Zhang, et al., 2010; Zhang, et al., 2014; Liu, et al., 2017) and, thus, report changes in PaO_2_ as well. Although the slope of minute ventilation vs. SaO_2_ was significantly smaller in the elderly women than men, there was no difference in the slopes of minute ventilation vs. P_ET_O_2_ between the sexes, This discrepancy is probably related to a non-simply linear decrease in P_ET_O_2_ during hypoxia (Figure 2D). The study sample size is small, especially in the male group (n = 7), since this study is not *a priori* in design. Moreover, since cardiorespiratory fitness or function may affect exercise-induced arterial hypoxemia, future studies with expanded sample size are required to define potential fitness-related differences in hypoxia-induced arterial hypoxemia in elderly men and women.

Despite these limitations, there is an important practical implication of this study: that the sex-related differences should be taken into account when applying normobaric poikilocapnic IH training as a *non-pharmacological* prophylactic and therapeutic strategy to improve neurocognitive performance, physiological function or physical well-being in elderly men and women. In elderly men, breathing moderately hypoxic air containing ≥10% O_2_ for ≤3 min may barely attain the threshold to trigger compensatory responses based on active cerebrovasodilation (Liu, et al., 2017). Most elderly men can tolerate ≥6 min exposure to 10% O_2_ without experiencing severe hypoxemia. On the other hand, in some elderly women ≥6 min exposure to 10% O_2_ may reduce SaO_2_ and/or ScO_2_ to critical levels, i.e., *c*. ≤65% SaO_2_ or ≤50% ScO_2_ (Figure 1). Since more severe hypoxemia cannot further augment cerebral vasodilation and blood flow to compensate for cerebral tissue hypoxia (Liu, et al., 2017), it may induce dizziness or lightheadedness.

In conclusion, this study demonstrated more intense arterial hypoxemia in elderly women than elderly men during hypoxic breathing. Physiologically, this greater hypoxia-induced hypoxemia may be explained by an increase in O_2_-hemoglobin dissociation and attenuation of the chemoreflex-mediated ventilatory compensation in the elderly women vs. their age-matched male counterparts. These sex-related differences in arterial hypoxemia and the hypoxemia-elicited ventilatory responses should be taken into account when implementing hypoxic regimens for elderly adults.

## Data Availability

The raw data supporting the conclusions of this article will be made available by the authors, without undue reservation.
